# SPRING‐RIO TSE: 2D T_2_‐Weighted Turbo Spin‐Echo brain imaging using SPiral RINGs with retraced in/out trajectories

**DOI:** 10.1002/mrm.29210

**Published:** 2022-04-08

**Authors:** Zhixing Wang, Steven P. Allen, Xue Feng, John P. Mugler, Craig H. Meyer

**Affiliations:** ^1^ Department of Biomedical Engineering University of Virginia Charlottesville Virginia USA; ^2^ Department of Electrical and Computer Engineering Brigham Young University Provo Utah USA; ^3^ Department of Radiology & Medical Imaging University of Virginia Charlottesville Virginia USA

**Keywords:** fast imaging, neuroimaging, spiral imaging, T_2_‐weighted imaging

## Abstract

**Purpose:**

To develop a new approach to 2D turbo spin ‐echo (TSE) imaging using annular spiral rings with a retraced in/out trajectory, dubbed “SPRING‐RIO TSE”, for fast T_2_‐weighted brain imaging at 3T.

**Methods:**

A long spiral trajectory was split into annular segmentations that were then incorporated into a 2D TSE acquisition module to fully exploit the sampling efficiency of spiral rings. A retraced in/out trajectory strategy coupled with spiral‐ring TSE was introduced to increase SNR, mitigate T_2_‐decay induced artifacts, and self‐correct moderate off‐resonance while maintaining the target TE and causing no scan time penalty. Model‐based k‐space estimation and semiautomatic off‐resonance correction algorithms were implemented to minimize effects of k‐space trajectory infidelity and B_0_ inhomogeneity, respectively. The resulting SPRING‐RIO TSE method was compared to the original spiral‐ring (abbreviated “SPRING”) TSE and Cartesian TSE using simulations, and phantom and in vivo acquisitions.

**Results:**

Simulation and phantom studies demonstrated the performance of the proposed SPRING‐RIO TSE pulses sequence, as well as that of trajectory correction and off‐resonance correction. Volunteer data showed that the proposed method achieves high‐quality 2D T_2_‐weighted brain imaging with a higher scan efficiency (0:45 min/14 slices versus 1:31 min/14 slices), improved image contrast, and reduced specific absorption rate compared to conventional 2D Cartesian TSE.

**Conclusion:**

2D T_2_‐weighted brain imaging using spiral‐ring TSE was implemented and tested, providing several potential advantages over conventional 2D Cartesian TSE imaging.

## Abstract 2

Click here for author‐reader discussions

## 1 INTRODUCTION

T_2_‐weighted pulse sequences are widely used for clinical neuroimaging because of their high sensitivity for many neurological disorders. Turbo spin‐echo (TSE) pulse sequences, also known as fast spin‐echo (FSE), are commercial implementations of the Rapid Acquisition with Relaxation Enhancement (RARE) technique,^1^ and have replaced conventional spin‐echo (SE) technique for T_2_‐weighted imaging, due to their faster acquisition times. Therefore, 2D and 3D TSE have become the workhorse pulse sequences for T_2_‐weighted imaging in the routine clinical MR neuro exam.^2^, ^3^

Although the 2D Cartesian TSE sequence is one of the standard methods for T_2_‐weighted imaging, the high specific absorption rate (SAR) induced by a large number of refocusing RF pulses limits its use at high magnetic fields. The long RF pulse train may produce T_2_‐decay blurring^4^, ^5^ and may also alter the image contrast compared to the conventional SE.^6^ Another limitation of Cartesian TSE is a relatively long scan time attributed to the low sampling efficiency, typically taking minutes for images with sub‐millimeter spatial resolution, which may induce motion artifacts from patient motion.

Compared to Cartesian sampling, spiral imaging^7^ covers k‐space more efficiently via a higher average k‐space velocity, thereby reducing total scan time and/or improving SNR. Spiral imaging also has the advantage of reduced sensitivity to motion artifacts, and flow artifacts are often minimal and isotropic. Spiral acquisitions have been incorporated into a 2D TSE signal generation module via two strategies: an interleaved, rotated spiral‐arm segmentation and an annular ring segmentation. The first strategy, as proposed by Li et al.,^8^ shows that this spiral‐based TSE technique offers advantages over conventional Cartesian TSE in terms of SNR efficiency, improved image contrast, and reduced SAR. However, this method requires a double‐encoding strategy and a signal‐demodulation method to mitigate swirl‐like artifacts due to T_2_‐decay induced signal variation, extending the scan time. The annular ring strategy^9^, ^10^, ^11^, ^12^, ^13^ splits long spiral trajectories into several annular segments, with the benefit of reduced T_2_‐decay artifacts by converting the T_2_‐dependent signal modulation into a k‐space apodizing filter. This method was first implemented in abdominal imaging within one breath‐hold^9^ and single‐shot brain imaging,^10^ showing promising potential for fast T_2_‐weighted imaging.

Previously, we described a 2D spiral‐ring (abbreviated “SPRING”) TSE technique^11^ which was adapted from the method proposed by Block et al.^9^ for dual‐contrast T_2_‐weighted imaging at 1.5T using a spiral ring segmentation and a shared‐view acquisition. The results demonstrated that ring segmentation leads to a smoothed T_2_‐dependent weighting of signal amplitudes across k‐space and thus benign artifact behavior. One key advantage of this annular‐ring segmentation, compared to the interleaved, rotated spiral‐arm segmentation, is that there is no need for the double‐encoding strategy, thus resulting in a shorter scan time. However, there are still challenges associated with this technique, such as residual T_2_‐decay blurring and off‐resonance induced signal loss. In addition, early echoes are typically discarded to achieve the desired T_2_‐weighting, resulting in a reduced scan efficiency. Furthermore, the annular‐ring sampling strategy has not been fully explored for brain imaging via either a single‐shot excitation or multi‐shot acquisitions.

In this study, a new approach to 2D TSE imaging using annular spiral rings with a retraced in/out trajectory, dubbed “SPRING‐RIO TSE^12^”, is proposed to address these aforementioned challenges. First, we introduce the sampling strategy of annular rings with retraced in/out (RIO)^12^, ^13^, ^14^ segments and demonstrate potential advantages of this approach via simulations. Second, we describe methods for correcting for k‐space trajectory infidelity and off‐resonance effects. Finally, we validate the feasibility of the proposed technique and compare its performance to that of SPRING TSE and Cartesian TSE in phantom and in vivo scans.

## 2 METHODS

### 2.1 Technique

#### 2.1.1 Pulse sequence

SPRING TSE^9^, ^11^ has high acquisition efficiency, but is prone to off‐resonance induced artifacts and signal loss because the center of k‐space is not aligned with the spin echo, and the phase change does not grow linearly with k‐space radius due to the interspersed refocusing RF pulses. Furthermore, although the k‐space apodizing filter introduced by annular‐ring acquisition can mitigate the T_2_‐decay artifacts by smoothing the signal modulation along the echo train, this filter inevitably leads to an apparent spatial resolution loss.

**FIGURE 1:**
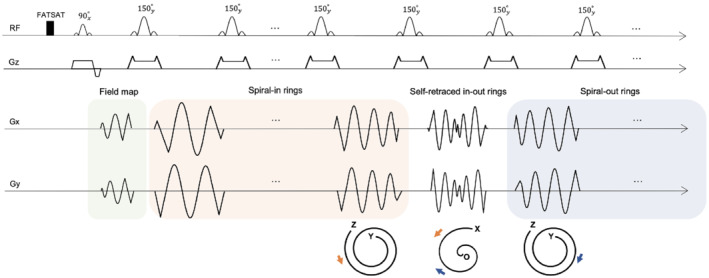
Pulse sequence timing diagram showing the sampling strategy, which includes fat saturation, field‐map acquisition, and data acquisition using annular spiral rings. The center of k*‐*space is sampled by a self‐retraced spiral in‐out (X – O – X) arm. The spiral‐in rings (Z – Y) in the orange box are designed to cover the outer portion of k‐space, while the spiral‐out (Y – Z) rings in the blue box retrace the corresponding spiral‐in rings. The X point in the inner self‐retraced in‐out rings is sampled twice in k‐space, and the neighboring Y point is sampled in both the preceding spiral‐in ring and the following spiral‐out ring. For each shot, the number of spiral‐in rings (including the first half of the central spiral‐in/out ring) is equal to that of spiral‐out rings (including the latter half of the central spiral‐in/out ring), which was set to 7, with a total of 15 shots per measurement. The refocusing RF pulse angles are set to 150° for reduced SAR

To mitigate image artifacts and blurring, and to further improve the sampling efficiency, the SPRING‐RIO TSE pulse sequence is proposed as follows. The pulse sequence timing diagram depicted in Figure 1 shows the sampling strategy, which includes fat saturation to suppress lipid signals, field map acquisition, and TSE data acquisition using annular spiral rings. Short spiral‐out arms were placed in the interval between the excitation and the first refocusing RF pulses for field map acquisition, with a 1 ms interval between the odd and even shots, to allow for a range of ±500 Hz off‐resonance. The TSE data were then collected by a series of spiral rings, including a self‐retraced spiral in‐out ring for the center of k‐space, and spiral‐out rings at the end of the echo train paired with time‐reversed, spiral‐in rings with opposite gradient polarity at the beginning of the echo train, to acquire the outer portion of k‐space. Examples of retraced spiral trajectories are shown at the bottom of Figure 1. The inner portion of k‐space was sampled along the path X – O – X in a single echo spacing (ESP), while the outer k‐space was sampled twice via the path Z – Y by the earlier spiral‐in rings and the path Y – Z by the later spiral‐out rings. A given ring of k‐space values was sampled by two trajectories, $k_{j,p}\left(t\right)$ and $k_{j,q}\left(t\right)$, for which the subscripts j,p and j,q stand for the same $j^{\mathrm{th}}$ k‐space ring coverage but acquired at two different echoes p and q, by the respective spiral‐out rings and the spiral‐in rings. $T_{p}$ and $T_{q}$ refer to the time interval between the excitation RF pulse and the center of the readout window at $p^{\mathrm{th}}$ and $q^{\mathrm{th}}$ echoes, respectively. Because of symmetry of the retracing about TE,

(1) $$T_{p}+T_{q}=2\;\mathrm{TE}$$

where 0≤p≤L−1,−L+1≤q≤0, and L is the total number of spiral‐out rings.

For $t\in \left[-\frac{T}{2},\frac{T}{2}\right]$, $k_{j,p}\left(t\right)$ and $k_{j,q}\left(t\right)$ can be written as:

(2) $$k_{j,p}\left(t\right)=k_{j,q}\left(-t\right)=-k_{j,q}\left(t\right)$$

where T is the readout time, and $k_{j,p}\left(t\right)$ and $k_{j,q}\left(t\right)$ are constrained to be antisymmetric and mirrored at time points symmetric about the spin echo. The central self‐retraced spiral in‐out segment can be considered as the special case when p=q=0.

#### 2.1.2 Gradient design

In a typical TSE acquisition module, the gradient‐induced dephasing within each echo spacing should be constant, and non‐zero, to preserve the Carr‐Purcell‐Meiboom‐Gill (CPMG) condition.^15^ For our design, a constant non‐zero zeroth order gradient moment is provided by crusher gradients surrounding the refocusing RF pulses. The zeroth order gradient moments of the spiral readout gradients were nulled in each echo spacing by surrounding them with prephaser gradients that move out from the origin of k‐space to the beginning of the segment, and rephaser gradients that move back to the origin from the end of the segment. The prephaser and rephaser lobes were designed to be played simultaneously with the crusher gradients. To obtain submillimeter in‐plane spatial resolution, an interleaved multi‐shot acquisition was used to interleave the spiral ring gradients over several repetition times to cover all of k‐space. A constant density spiral trajectory design was used, based on the algorithm of Meyer et al.,^7^, ^16^ to obtain minimum‐time spiral readouts constrained by gradient slew rate and amplitude limits.

The spiral gradient waveforms for multi‐shot SPRING‐RIO TSE were designed using the following six‐step procedure.

- a single, very long spiral‐out arm was generated with desired imaging properties, such as FOV, spatial resolution, and number of shots;
- this spiral arm was split into L segments of equal time duration;
- the gradient polarity of a copy of the first segment was inverted, and this segment was then time‐reversed and placed in front of the original first segment to generate a self‐retraced spiral‐in‐out annular ring, which is played at the effective TE (${\mathrm{TE}}_{\mathrm{eff}}$);
- the remaining second to L segments were placed consecutively at the subsequent TSE echoes (following ${\mathrm{TE}}_{\mathrm{eff}}$);
- the gradient polarities of copies of the second to L segments were inverted, and these segments were then time‐reversed and placed consecutively, in reverse order, at the TSE echoes preceding ${\mathrm{TE}}_{\mathrm{eff}}$;
- the waveforms generated as described in steps 1–5 were rotated N times to obtain a total of N*(2*L−1) spiral ring waveforms.

For multi‐shot SPRING TSE, L gradient segments were generated using steps 1 and 2 above. Then, the L segments were placed sequentially at the TSE echoes, starting with the first segment played at ${\mathrm{TE}}_{\mathrm{eff}}$. Finally, the waveforms were rotated N times to obtain a total of N*L spiral ring waveforms.

The selection of L depends on the in‐plane spatial resolution, FOV, the readout acquisition time, and the total scan time. For example, for a given FOV, spatial resolution, and the total scan time, SPRING‐RIO TSE with a longer readout window per ring requires a smaller value of L, and vice versa. Sequences with a larger L will reduce the sensitivity to B_0_ inhomogeneities but increase the RF SAR. The ${\mathrm{TE}}_{\mathrm{eff}}$ may also affect the selection of L for SPRING‐RIO TSE, because $L\times \mathrm{ESP}\approx {\mathrm{TE}}_{\mathrm{eff}}$, if L fully retraced spiral‐in rings are placed in the early echoes. Empirically, sequences with a longer ESP and a smaller L will produce a shorter ${\mathrm{TE}}_{\mathrm{eff}}$ compared to that with a shorter ESP and a larger L, since the former one saves certain amounts of time, such as the time used for the refocusing RF. Here, L=7 and N=15 were chosen for both multi‐shot SPRING TSE and SPRING‐RIO TSE.

#### 2.1.3 k‐Space trajectory fidelity

In non‐Cartesian readout sequences such as spiral imaging, eddy currents and anisotropic delays of the gradient system generally affect the fidelity of the k‐space trajectory and cause image blurring and/or artifacts^17^ if not corrected. It is possible to measure the actual k‐space trajectory in a calibration measurement and use this measured trajectory for image reconstruction.^18^ However, it is impractical to measure the actual trajectory for a wide variety of different acquisitions, since the actual trajectory varies depending on spiral parameters, and the calibration measurement is time consuming. In this work, we applied a model‐based method,^19^, ^20^ which has been studied for spiral‐out and spiral‐in/out sequences, to estimate the actual trajectory for each annular spiral ring. The modified k‐space trajectory estimation model as introduced by Feng et al.^20^ is:

(3) $$\tilde{k}\left(t\right)\approx \left(1+A\right)k_{d}\left(t\right)+B\int_{0}^{t}k_{d}\left(\tau \right)d\tau ,$$

where $\tilde{k}\left(t\right)$ is the estimated k‐space trajectory, $k_{d}\left(t\right)$ is the k‐space trajectory on one physical axis with a gradient delay ∆T, and *A* and *B* are assumed to be constant values and independent of the image orientation and spiral parameters. To determine the values of the optimal delay time ∆T, *A*, and *B* on each physical axis, a set of trajectory measurements was performed on the scanner using Duyn's method,^18^ followed by a least‐squares fit with the model given in Equation (3).

To evaluate the effects of the k‐space trajectory estimation model, we measured the actual k‐space trajectory in phantom experiments for SPRING TSE and SPRING‐RIO TSE sequences. Both the estimated k‐space trajectories themselves and the images reconstructed with these trajectories were compared to the theoretical trajectories and the corresponding images.

### 2.2 Simulations

All simulations were implemented in MATLAB (R2020b software; MathWorks, Natick, MA). To demonstrate the benefits of the RIO trajectory design of SPRING‐RIO TSE, we simulated its response to system nonidealities, T_2_‐decay effects, and B_0_ off‐resonance effects, and compared the results to those of SPRING TSE. A few properties of these two k‐space trajectories must be defined (see below) before simulations. Assuming T_2_*‐decay effects during each readout are negligible when compared to T_2_‐decay effects along the echo train, the received MR signal for the $j^{\mathrm{th}}$ k‐space ring acquired at the echo time $T_{p}$ can be modeled as below:

(4) $$s_{j,p}\left(t\right)=\int m\left(r\right)\;e^{-i2\pi k_{j,p}\left(t\right)r}e^{-i\omega \left(r\right)t}\;e^{\frac{-T_{p}}{T_{2}}}dr$$

where m(r) is the object's complex‐valued magnetization and ω(r) is the spatially varying resonant frequency of the object. Equation (4) describes the signal for SPRING TSE during one ring acquisition. It is shown in Supporting Information Appendix A, which is available online that, for $t\in \left[-\frac{T}{2},\frac{T}{2}\right]$, the signal resulting from averaging the data from a retraced in‐out trajectory of SPRING‐RIO TSE can be written as:

(5) $$\begin{array}{cc} s\left(t\right) & =\int m\left(r\right)\;e^{-i2\pi k_{j,p}\left(t\right)r}e^{\frac{-\mathrm{TE}}{T_{2}}}\left[\cos\left[\omega \left(r\right)t\right]\cosh\left(\frac{T_{p}-\mathrm{TE}}{T_{2}}\right)\right.\\ & \;+\left.\text{isin}\left[\omega \left(r\right)t\right]\sinh\left(\frac{T_{p}-\mathrm{TE}}{T_{2}}\right)\right]\mathrm{dr}. \end{array}$$

#### 2.2.1 T_2_ ‐Decay effects

Ignoring B_0_ inhomogeneity and T_2_* relaxation during the acquisition window, but including T_2_ relaxation along the echo train direction, we can simplify Equations (4) and (5) for SPRING TSE and SPRING‐RIO TSE as below:

(6) $$s_{\text{SPRING}}\left(t\right)=\int m\left(r\right)\;e^{-i2\pi k_{j,p}\left(t\right)r}\;e^{\frac{-T_{p}}{T_{2}}}dr$$

(7) $$\begin{array}{cc} & s_{\text{SPRING}-\mathrm{RIO}}\left(t\right)\\ & =\int m\left(r\right)\;e^{-i2\pi k_{j,p}\left(t\right)r}\;e^{\frac{-\mathrm{TE}}{T_{2}}}\;\cosh\left(\frac{T_{p}-\mathrm{TE}}{T_{2}}\right)dr. \end{array}$$

The impact of T_2_ relaxation was calculated and compared among each of the described trajectories. A matrix of ones was inverse‐gridded with each trajectory, and T_2_ relaxation on the order of the k‐space radius was simulated by exponentially decreasing the amplitude of the simulated data. The simulated data were then gridded and displayed as the windowing patterns of k‐space. Point‐spread‐functions (PSFs) were calculated with zero padding and normalized to [0,1], and the corresponding full width at half maximum (FWHM) values were compared to determine the effects of T_2_ decay on these sampling trajectories. Moreover, digital phantom simulations for SPRING TSE and SPRING‐RIO TSE were performed, and the corresponding mean structural similarity indices (SSIM)^21^ were calculated and compared based the reference condition without T_2_ decay. The mean SSIM is defined as below:

(8) $$\begin{array}{cc} & \text{mean}\;\text{SSIM}\left(X,Y\right)\\ & \;=\frac{1}{M}\sum_{j=1}^{M}\frac{\left(2\;u_{x_{j}}u_{y_{j}}+C_{1}\right)\left(2\;\sigma_{x_{j}y_{j}}+C_{2}\right)}{\left({u_{x_{j}}}^{2}+{u_{y_{j}}}^{2}+C_{1}\right)\left({\sigma_{x_{j}}}^{2}+{\sigma_{y_{j}}}^{2}+C_{2}\right)}\;, \end{array}$$

where X and Y are two input images, $x_{j}$ and $y_{j}$ are the image contents at the $j^{\mathrm{th}}$ local window, M is the number of local windows in the image. u is the mean intensity, and σ is the standard deviation (SD) over one local window. $C_{1}={\left(0.01*L\right)}^{2}$, and $C_{2}={\left(0.03*L\right)}^{2}$ are used here as the default parameters, where L is the dynamic range of the images. The maximum mean SSIM index value 1 is achieved only if X and Y are identical. For these simulations, FOV = 230 mm, echo train length (ETL) = 7 (SPRING TSE) or 13 (SPRING‐RIO TSE), ESP = 13.5 ms, and T_2_ = 70 ms.

#### 2.2.2 B_0_ Off‐resonance effects

The k‐space phase of an off‐resonant point object in SPRING‐based TSE acquisitions does not grow monotonically with increasing k‐space radius.^9^ Instead, phase is accrued from off‐resonance over each echo spacing, with a (refocused) zero phase at the center of each echo spacing and a phase at the beginning of the next echo spacing that is inverted compared to that at the end of the preceding echo spacing.

To assess the extent of off‐resonance effects, PSFs for SPRING TSE and SPRING‐RIO TSE trajectories with various amounts of off‐resonance were simulated by performing nonuniform fast Fourier transform (NUFFT) reconstruction on a matrix of ones. Off‐resonance was added by linearly increasing the phase of the simulated data during each echo spacing. The corresponding digital phantom images with three different amounts of phase accumulated at the end of the readout were further simulated for visual comparison between these two sequences. SSIM values were calculated and compared as well. For these simulations, FOV = 230 mm, ETL = 7 (SPRING TSE) or 13 (SPRING‐RIO TSE), ESP = 15 ms, ADC = 8 ms, and offset frequency = 31.25 Hz (1/4 cycles), 62.5 Hz (1/2 cycles) or 93.75 Hz (3/4 cycles).

### 2.3 MRI experiments

#### 2.3.1 Data acquisition

Experiments were performed on a 3T scanner (MAGNETOM Prisma, Siemens Healthcare, Erlangen, Germany) with a 32‐channel head coil.

In a phantom study, axial data from a resolution phantom were acquired with SPRING TSE and the proposed SPRING‐RIO TSE to evaluate the efficacy of the RIO trajectory design. Model‐based trajectory measurements were performed for both sequences, and the estimated trajectories were then compared to the nominal trajectories in terms of image quality such as edge artifacts and blurring to demonstrate the necessity of trajectory infidelity correction. Relevant spiral imaging parameters include FOV = $180\times 180\;{\mathrm{mm}}^{2}$, spatial resolution $=0.60\times 0.60\;{\mathrm{mm}}^{2}$, slice thickness = 4 mm, refocusing RF flip angle $=150^{{}^{\circ}}$, ETL = 7 for SPRING TSE and 13 for SPRING‐RIO TSE, ESP = 14.8 ms with ADC = 7 ms. In k‐space trajectory measurements, the distance between the excited slice and the isocenter was 35 mm, and the slice thickness was 0.6 mm.

Five healthy volunteers with informed consent participated in this study and were scanned using the two spiral‐based TSE sequences and standard Cartesian TSE to evaluate the overall image quality. For each of these three sequences, data were acquired consecutively at the same image planes with 14 slices, 4 mm slice thickness, and 2 mm gap. Axial, coronal, and sagittal slices of the head were collected, with the FOV set to $230\times 230\;{\mathrm{mm}}^{2}$ for the axial plane, increasing to $250\times 250\;{\mathrm{mm}}^{2}$ for coronal and sagittal planes with slightly reduced resolution to avoid aliasing. The data of each slice were acquired twice for SPRING TSE and SPRING‐RIO TSE sequences, with 45 s per measurement; therefore, one signal average (1‐NSA) requires 0:45 min total scan time while two signal averages (2‐NSA) require 1:30 min. Spiral k‐space trajectories were estimated based on the system parameters obtained from the model‐based trajectory calibration. For all sequences, a fat saturation pulse was used to null the bright fat signal and avoid the strong chemical shift effect at 3 T, and the refocusing RF flip angle was set to $150^{{}^{\circ}}$, which was used to reduce SAR to an acceptable value for Cartesian TSE. Supporting Information Table S1 lists additional parameters of these three pulse sequences.

#### 2.3.2 Image reconstruction

The reconstruction was performed offline in MATLAB. The NUFFT code from the Michigan Image Reconstruction Toolbox (MIRT) package^22^ was used for direct 2D non‐Cartesian image reconstruction. Coil sensitivity maps were computed from the center k‐space data of the field map using ESPIRiT.^23^ To illustrate the performance of the trajectory correction, phantom images were reconstructed and compared with the nominal k‐space trajectory and the estimated k‐space trajectory.

For spiral imaging with long readouts, deblurring is an essential step to correct for off‐resonance‐induced phase errors. There have been many different deblurring techniques proposed for non‐Cartesian off‐resonance correction.^24^, ^25^, ^26^, ^27^, ^28^, ^29^, ^30^, ^31^ Most of the deblurring methods are based on knowledge of a field map, which can be derived from an additional 2‐TE gradient recalled echo scan^24^, ^25^, ^26^, ^27^ or can be estimated directly or partially from the image itself using the techniques termed “automatic^28^, ^29^” or “semiautomatic” deblurring.^30^, ^31^ In this work, semiautomatic deblurring of the component images using an established minimized phase objective function^28^, ^29^, ^30^ was applied to SPRING TSE, with the objective function:

(9) $$\min_{\omega_{i}}\int h\left(r-r^{\prime }\right)\;{\left|\text{Imag}\left\{\tilde{m}(r^{\prime };\omega_{i}\left(r^{\prime }\right))\right\}\right|}^{\alpha }dr^{\prime },$$

where α takes on values in the range from 0.5 to 1, Imag is the imaginary part of the image, $\tilde{m}\left(r;\omega_{i}\left(r\right)\right)$ is the image reconstructed at demodulation frequency $\omega_{i}$, and h(r) is the convolution kernel chosen to be a circularly symmetric Gaussian window.

Regarding SPRING‐RIO TSE, as noted by Fielden et al.^14^ and Allen et al.,^31^ moderate off‐resonance effects can be automatically corrected by the RIO design; however, at large off‐resonance values, this effect quickly degrades, and substantial blurring may remain. We chose the semiautomatic deblurring method with a maximized energy objective function proposed by Allen et al.^31^ for a specific RIO trajectory in spiral imaging and extended it to correct for off‐resonance effects in SPRING‐RIO TSE, using the objective function:

(10) $$\max_{\omega_{i}}\int h\left(r-r^{\prime }\right)\;\tilde{m}\left(r^{\prime };\omega_{i}\left(r^{\prime }\right)\right)\;\tilde{m}{\left(r^{\prime };\omega_{i}\left(r^{\prime }\right)\right)}^{*}dr^{\prime },$$

where $\tilde{m}{\left(r;\omega_{i}\left(r\right)\right)}^{*}$ is the complex conjugate of $\tilde{m}\left(r;\omega_{i}\left(r\right)\right)$. Supporting Information Appendix B shows that the global maximum of Equation (10) is invariant with T_2_ decay and invariant with image phase, which eliminates the need to accurately remove the incidental phase before applying this criterion.

A low‐resolution field map for the semiautomatic deblurring method was generated from the first short spiral‐out arms by extracting the phase difference between the odd shots and even shots. Both phantom and in vivo brain images from SPRING TSE and SPRING‐RIO TSE were reconstructed and compared with and without deblurring methods.

#### 2.3.3 Image quality analysis

Quantitative evaluation of the proposed SPRING‐RIO TSE sequence and standard Cartesian TSE sequence was performed using phantom and in vivo brain data. For brain images, regions of interest (ROIs) were drawn in the gray matter (GM) and white matter (WM) on axial images from SPRING‐RIO TSE and Cartesian TSE acquisitions. Signal intensities were measured on five subjects, with 10 slices per subject, and the relative SNR of ROIs and image contrast between ROIs were then calculated. The apparent SNR was measured by dividing the mean image intensity in the specified region by the SD of the image intensity outside the phantom or skull and multiplying the result by the 0.66 Rayleigh distribution correction factor. Pairwise comparisons were performed on a total of 50 pairs of SNR measurements using the Tukey–Kramer method. Similarly, the apparent image contrast between ROIs was also measured using:

(11) $$\text{Contrast}=\frac{\left(\text{signal}\;1-\text{signal}\;2\right)}{0.5*\left(s\text{ignal}\;1+\text{signal}\;2\right)}$$

## 3 RESULTS

### 3.1 Simulations

**FIGURE 2:**
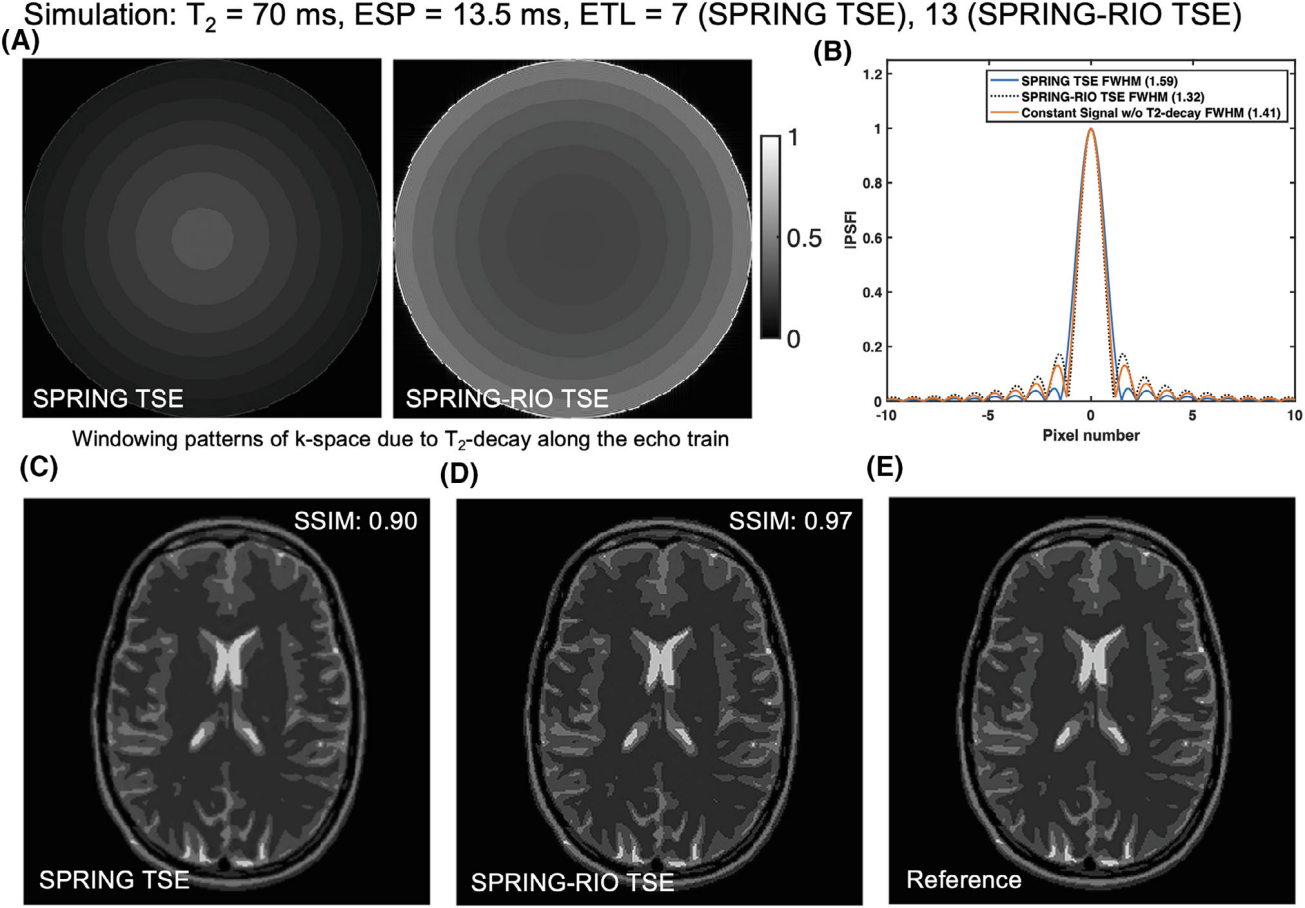
Simulation results of T_2_‐decay effect for SPRING TSE and SPRING‐RIO TSE. Windowing patterns of k‐space (A) and center lines of 2D PSFs (B) are shown for each trajectory. Note that the PSF was normalized to [0, 1] by dividing by its own peak; the peak for SPRING‐RIO TSE is higher than that for SPRING TSE because of the additional data acquired in the early echoes. Without a RIO scheme, the k‐space apodizing filter in SPRING TSE produces a broader PSF (FWHM:1.59), which leads to image blurring. SPRING‐RIO TSE produces a smoother frequency response, yielding a narrower main lobe of the PSF (FWHM:1.32), mainly because of a higher signal intensity acquired in the outer portion of k‐space. The SSIM value of SPRING‐RIO TSE (C) (0.97) versus that of SPRING TSE (D) (0.90) against Cartesian TSE (E) agrees well with the PSF calculations, demonstrating the benefits of RIO design for reducing T_2_‐decay induced blur and resolution loss

The simulation results illustrated the benefits of the RIO trajectory design of SPRING‐RIO TSE over the original SPRING TSE implementation in terms of T_2_‐decay induced resolution loss and off‐resonance induced artifacts and signal loss. The windowing patterns of k‐space due to T_2_ relaxation during acquisition are shown in the top row of Figure 2. For SPRING TSE, T_2_ relaxation results in a windowing of the data, with higher spatial frequencies losing signal, causing a broadening of the main lobe of the PSF (FWHM:1.59). The RIO strategy in SPRING‐RIO TSE produces a smoother frequency response, thus maintaining a PSF main lobe (FWHM:1.32) nearly as sharp as that for the constant signal with no T_2_‐decay effects (FWHM:1.41). The comparison among the bottom images reconstructed from SPRING TSE, SPRING‐RIO TSE, and the reference, and the corresponding SSIM values, demonstrate the advantage of using RIO sampling for reducing T_2_‐decay induced blur and subsequent resolution loss.

**FIGURE 3:**
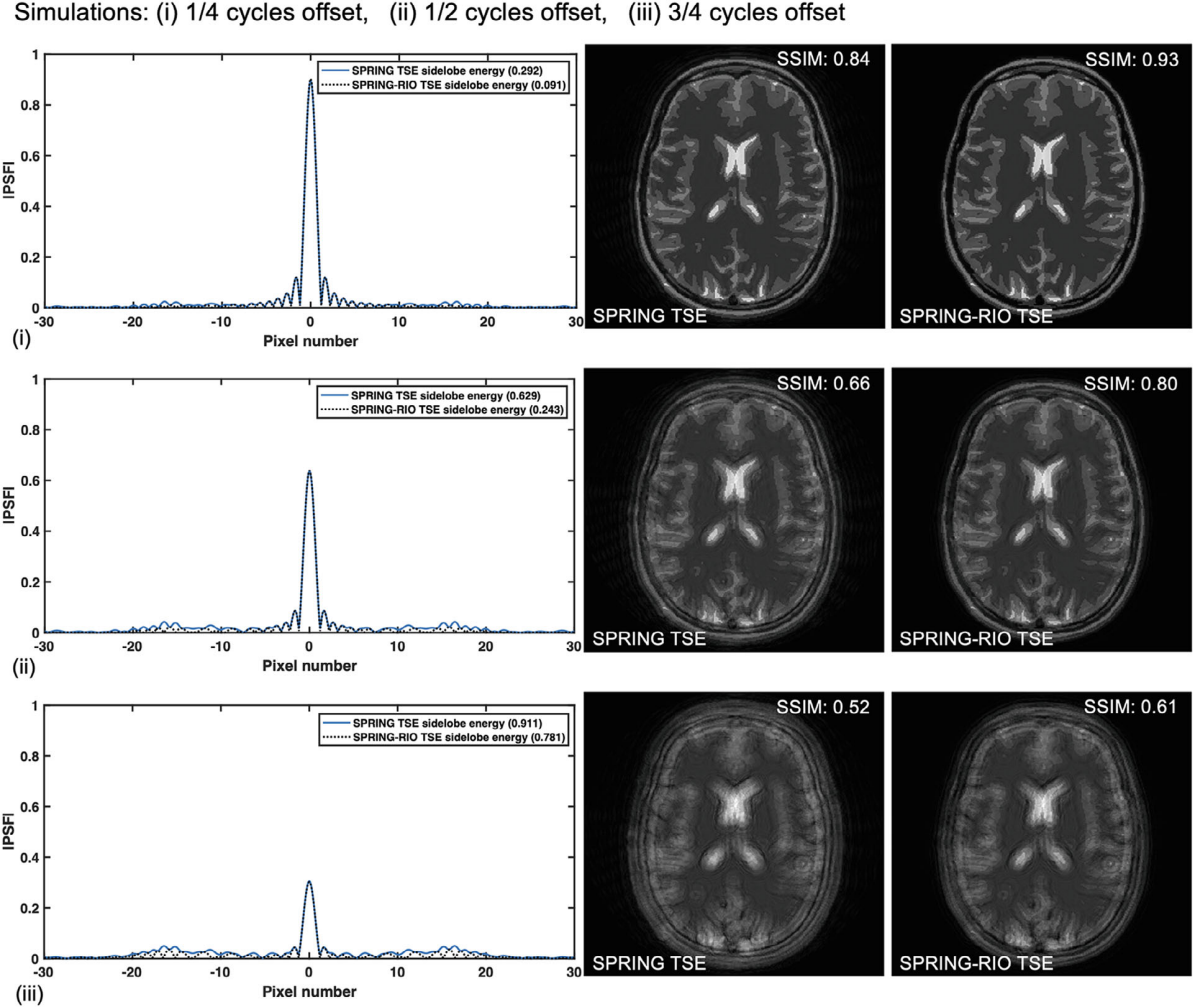
Simulation results of off‐resonance effects for SPRING TSE and SPRING‐RIO TSE. Off‐resonance effects were simulated for three different amounts (1/4, 1/2, and 3/4 cycles) of phase accumulation. Central lines of the 2D PSF and the side lobe energy of PSFs were calculated for each sequence variation. The PSF results show that the peak amplitudes of the main lobes for these two spiral‐ring based TSE sequences decrease with increasing off‐resonance frequency, causing signal loss yet without obvious loss in resolution. The digital brain image with no phase accumulation was used as the reference, and SSIM values were calculated between the reconstructed images of each sequence and the reference. Compared to SPRING TSE, the artifacts and signal loss in SPRING‐RIO TSE are reduced and largely self‐corrected when off‐resonance is moderate (i and ii)

Figure 3 shows the PSFs for 1/4, 1/2, and 3/4 phase cycles of phase accrual from off‐resonance over each of annular spiral ring segments. For both sequences, the effect of the refocusing RF pulses is to modulate the blurring so that there is not a substantial apparent loss of image resolution, but there is a signal loss that increases with off‐resonance frequency. SPRING‐RIO TSE performs better than SPRING TSE with less energy in the side lobes of the PSF. The reconstructed digital phantom images along with the SSIM values demonstrate this signal degradation with increasing off‐resonance frequency, and the merit of RIO trajectory design for self‐correction of moderate off‐resonance effects ranging up to 1/2 phase cycle. Difference images of simulated digital brain images from SPRING TSE or SPRING‐RIO TSE with T_2_ decay or off‐resonance effects compared to the reference are in Supporting Information Figure S1. Another simulation example of an inferior slice with air/susceptibility from a digital brain phantom with off‐resonance effects for SPRING TSE and SPRING‐RIO TSE can be found in Supporting Information Figure S2.

### 3.2 Phantom images

**FIGURE 4:**
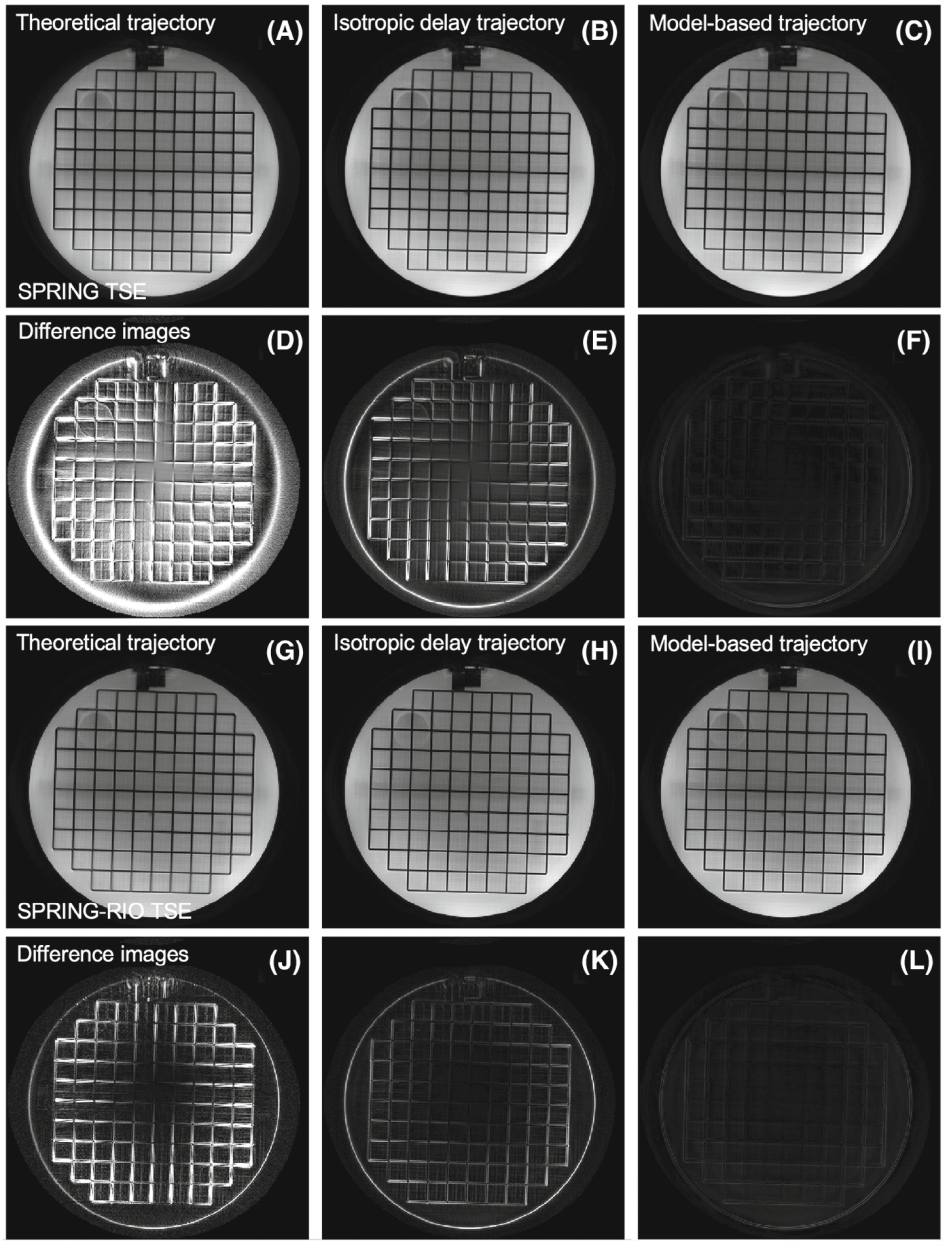
Reconstructed images of an axial slice in the resolution phantom from SPRING TSE (A‐F) and SPRING‐RIO TSE (G‐I), and absolute difference images relative to the goal images based on measured k‐space trajectories. The difference images are windowed to the same scale. A,G, Theoretical trajectory. B,H, Isotropic delay corrected trajectory. C,I, Model‐based corrected trajectory. The second and fourth rows show the difference images between the trajectory type immediately above and the goal image (e.g., (D) shows the difference between image (A) and the goal image)

Figure 4 shows reconstructions of one axial slice from a resolution phantom from SPRING TSE (Figure 4A–F) and SPRING‐RIO TSE (Figure 4G–I) acquisitions using the theoretical trajectory (Figure 4A,G), isotropic delay corrected trajectory (Figure 4B,H), and model‐based trajectory (Figure 4C,I), as well as their corresponding absolute difference images relative to the goal images based on measured k‐space trajectories. We observe that images with isotropic delay corrected k‐space trajectories still show noticeable artifacts, mainly around edges, and shading and shape distortions. A slight distortion remaining in k‐space trajectories (e.g., anisotropic delays and different eddy current terms on different physical gradient axes) would also cause significant artifacts. Improvements can be easily seen in Figure 4C,I when using a model‐based estimated trajectory, which removes most artifacts. Comparing difference images between SPRING TSE (Figure 4D,E) and SPRING‐RIO TSE (Figure 4J,K) sequences, the SPRING‐RIO TSE technique seems to be less sensitive to the gradient delays, most likely due to the time‐reversed signal average between spiral‐in and spiral‐out rings which averages some shape distortions, although this has not been fully explored.

**FIGURE 5:**
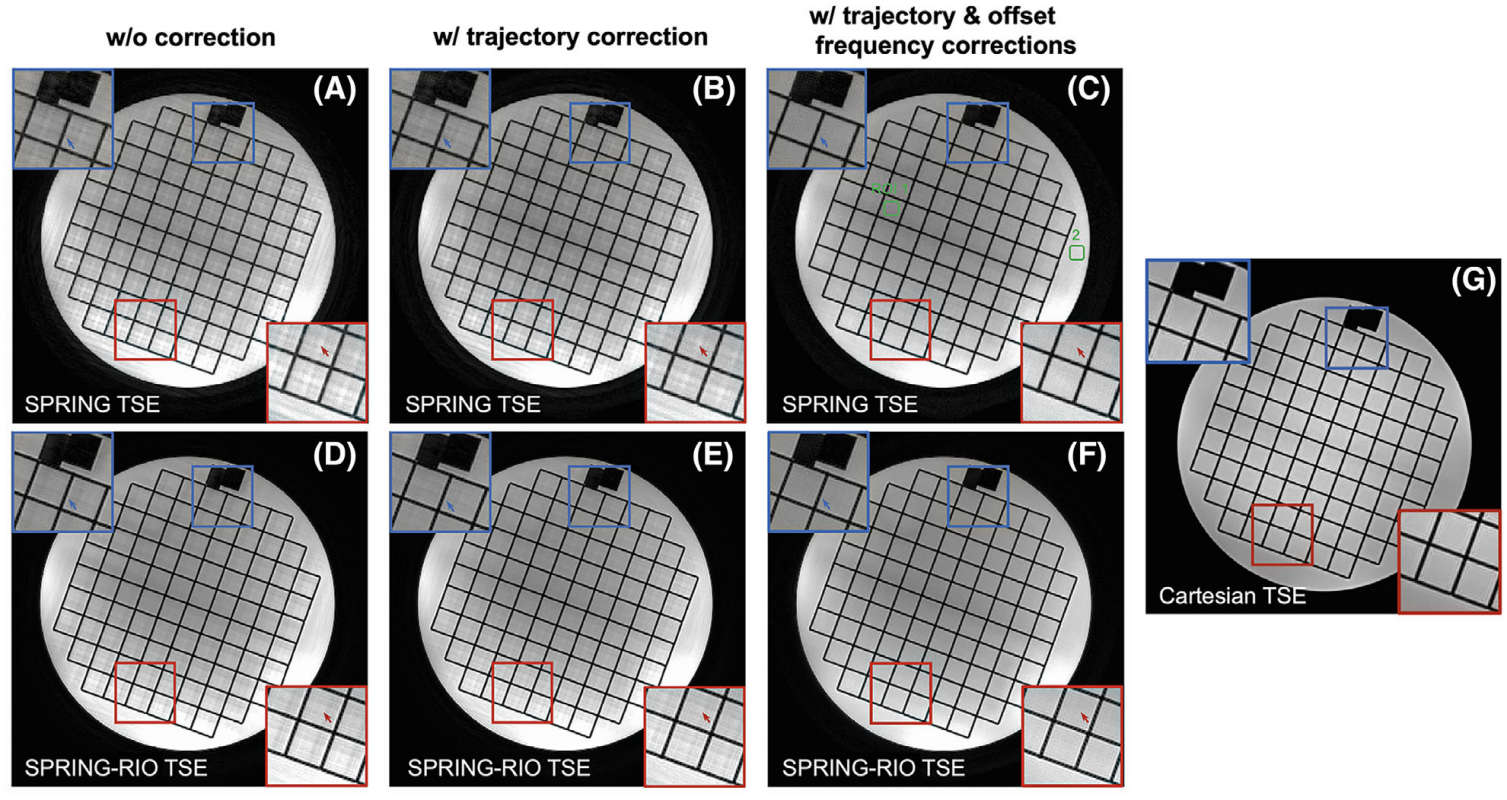
Performance of trajectory and off‐resonance corrections, and of the RIO scheme. A,D, The portions of the phantom highlighted by the blue and red boxes illustrate that, without correction, noticeable artifacts around edges, shading, and strong off‐resonance artifacts are present in the images. B,E, With trajectory correction, edge artifacts and shading are reduced (blue arrows). C,F, By further performing the off‐resonance correction, artifacts and signal loss are significantly reduced (red arrows). Comparing images A‐C and D‐F, higher SNR (ROI 1: 49 versus 69, ROI 2: 73 versus 86), fewer residual artifacts, and improved sharpness can be seen for SPRING‐RIO TSE (D‐F) than for SPRING TSE (A‐C). G, The image from Cartesian TSE is shown for reference

Figure 5 illustrates the efficacy of trajectory and off‐resonance corrections for SPRING TSE versus SPRING‐RIO TSE. The reduction of edge artifacts by using the model‐based estimated trajectories for both of the spiral‐ring sequences can be easily seen in Figure 5B,E from the zoomed portions of the images indicated by the boxes, when compared to the corresponding regions in Figure 5A,D. By further performing the aforementioned semiautomatic deblurring methods, both the artifacts and signal loss are significantly reduced in the fully corrected images shown in Figure 5C,F. Comparing images between SPRING TSE (Figure 5A–C) and SPRING‐RIO TSE (Figure 5D–F) sequences, images with higher SNR and improved sharpness can be seen for SPRING‐RIO TSE, primarily due to the additional spiral‐in rings acquired before the effective echo time. Furthermore, in the presence of nonlinear B_0_ variation, the uncorrected SPRING‐RIO TSE sequence presents fewer image artifacts than an uncorrected SPRING TSE acquisition (Figure 5B, E), thus demonstrating that the self‐correcting RIO trajectory shows certain robustness to moderate off‐resonance effects.

### 3.3 In vivo images

**FIGURE 6:**
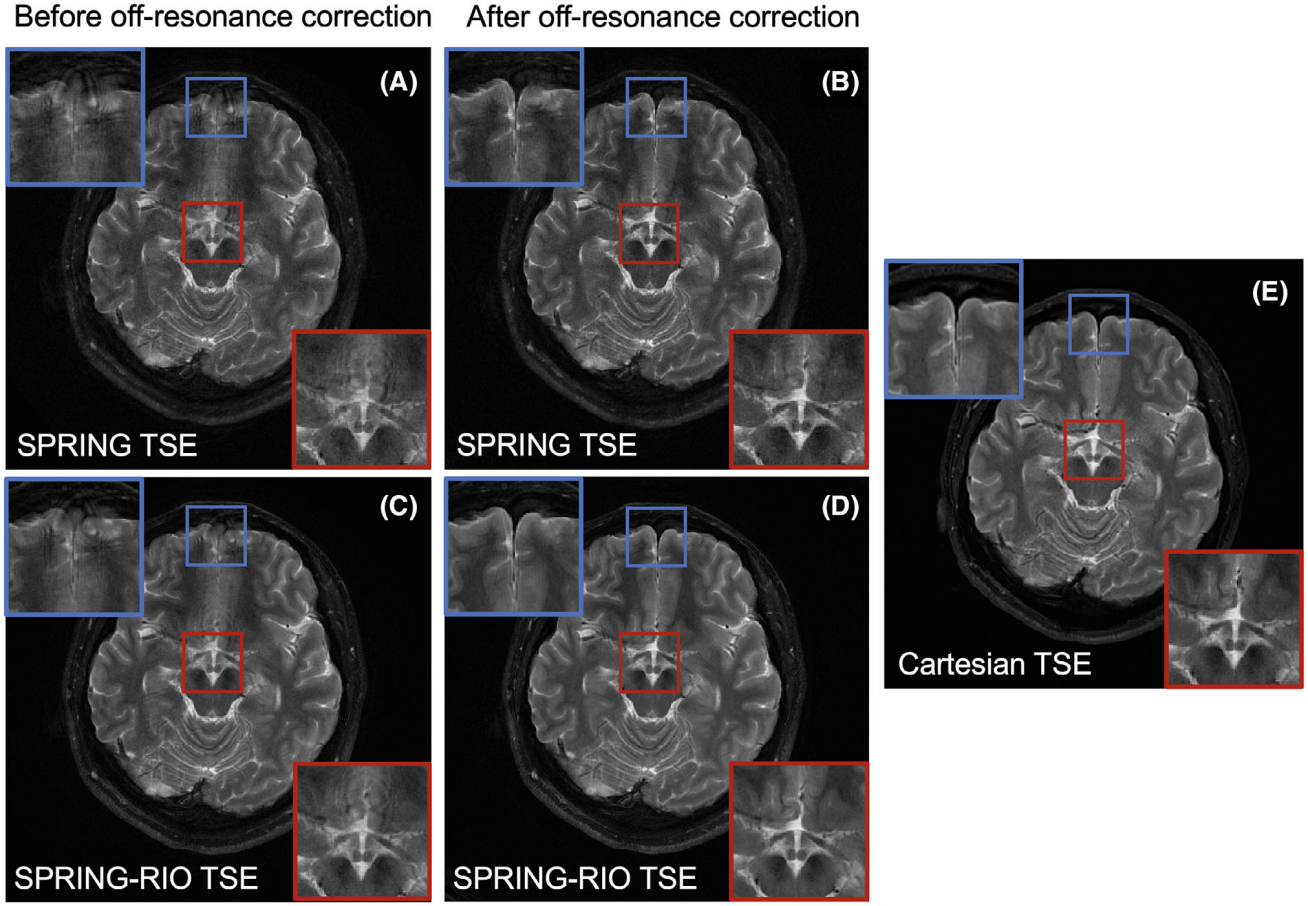
Comparison of axial brain images acquired with SPRING TSE (A,B) and SPRING‐RIO TSE (C,D), and reconstructed before (A,C) and after (B,D) off‐resonance correction. The images in the right column demonstrate the efficacy of off‐resonance correction. The SPRING‐RIO TSE acquisition with semiautomatic off‐resonance correction using maximized energy as a focusing criterion achieves overall better image quality than SPRING TSE acquisition with semiautomatic off‐resonance correction using minimum phase as a focusing criterion. E, The image from Cartesian TSE is shown for reference

Figure 6 displays axial brain images acquired with SPRING TSE and SPRING‐RIO TSE sequences and reconstructed before and after off‐resonance correction. The zoomed portions of the images on the left, before off‐resonance correction, are consistent with simulation results and phantom studies, showing that artifacts caused by modest B_0_ inhomogeneities can be reduced by the RIO design. The images in the right column demonstrate the efficacy of off‐resonance correction. The SPRING‐RIO TSE acquisition with semiautomatic off‐resonance correction using a maximized energy objective function achieves overall better image quality than the SPRING TSE acquisition with semiautomatic off‐resonance correction using a minimum phase objective function, in terms of SNR, residual artifacts, and image blurring.

**FIGURE 7:**
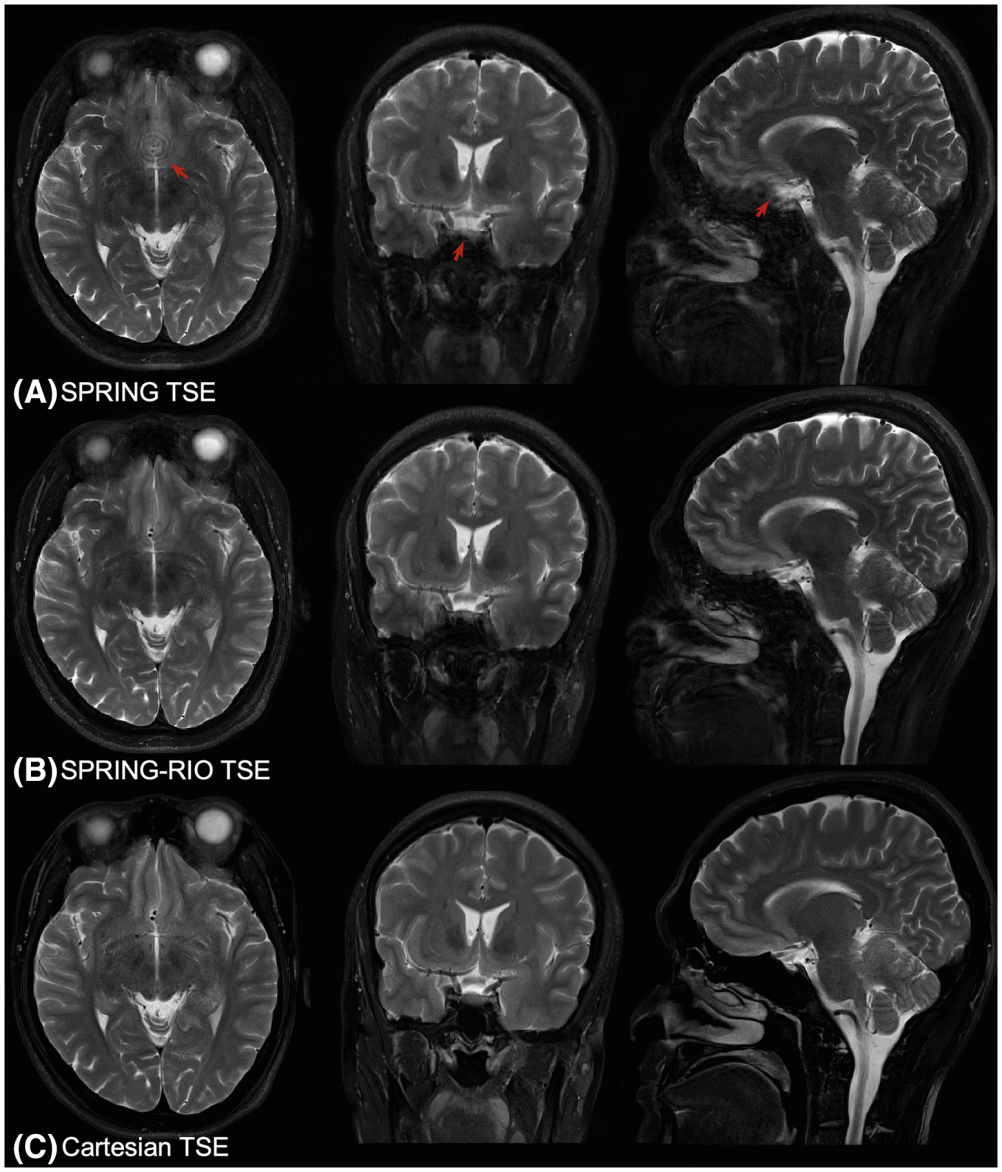
Comparison of trajectory‐ and off‐resonance‐corrected axial, coronal, and sagittal brain images from SPRING TSE (A) and SPRING‐RIO TSE (B). The red arrows point to regions where SPRING‐RIO TSE performs better than SPRING TSE, in terms of residual artifacts and image blurring. Tissues with short T_2_ values, such as skull and bone, present sharper details in SPRING‐RIO TSE than those in SPRING TSE. C, Images from Cartesian TSE are shown at the bottom for reference

Axial, coronal, and sagittal brain images from SPRING TSE and SPRING‐RIO TSE are shown in Figure 7. All the images are reconstructed using estimated trajectories and B_0_ off‐resonance corrections, and with two signal averages. Red arrows point to regions in the SPRING TSE brain images that show residual artifacts (presumably from off‐resonance) even after correction, especially near air‐tissue boundaries where the susceptibility gradients are relatively strong. Furthermore, we observe that, compared to SPRING TSE, SPRING‐RIO TSE produces sharper images with less T_2_‐decay induced blurring, as presented in some tissues with short T_2_ values, such as skull and bone.

**FIGURE 8:**
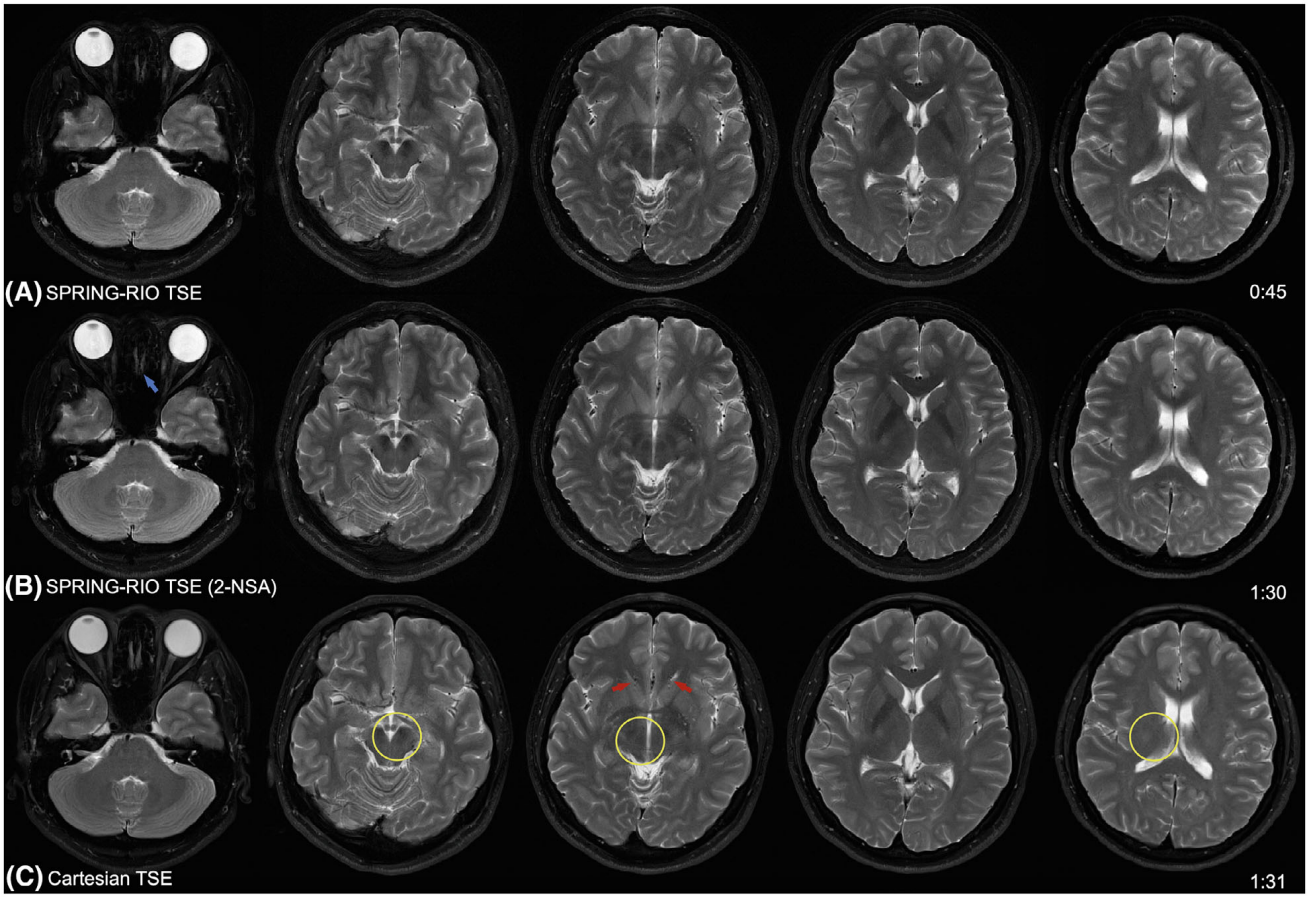
Comparison of in vivo axial images acquired using the proposed SPRING‐RIO TSE method and standard Cartesian TSE. From top to bottom are corrected images from SPRING‐RIO TSE with one signal average (A) and with two signal averages (B), and images from standard Cartesian TSE (C). The red arrows point to structures showing flow artifacts (left–right direction) from the anterior cerebral arteries in Cartesian TSE, while the blue arrow points to signal loss in SPRING‐RIO TSE. The yellow circles indicate regions where the image contrast is better in SPRING‐RIO TSE than in Cartesian TSE

**FIGURE 9:**
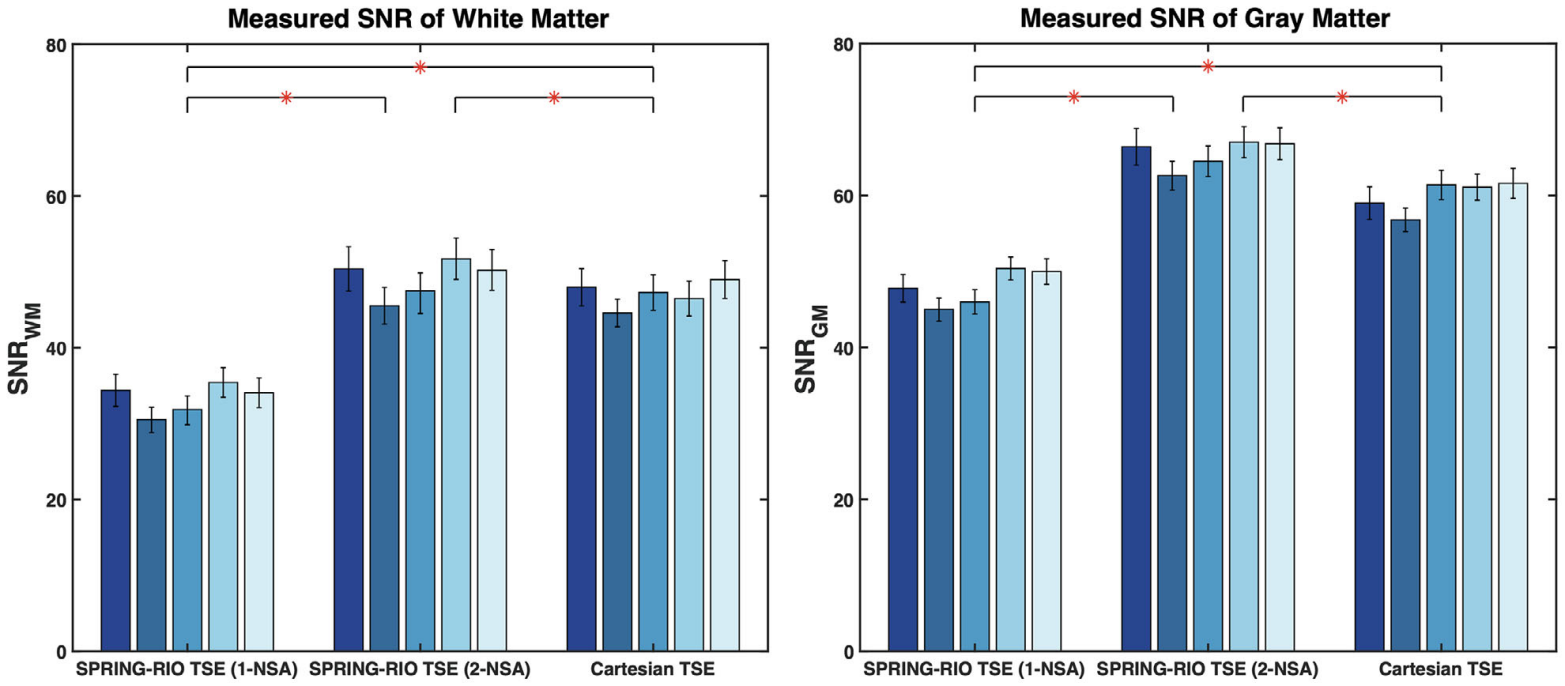
Measured SNR of ROIs in WM (left) and GM (right) with SPRING‐RIO TSE with 1‐NSA, with 2‐NSA, and standard Cartesian TSE. The different bars for each method represent the values computed for five different volunteers. For each volunteer, 10 slices are selected for SNR calculation, and thus pairwise comparisons among sequences are performed on a total of 50 pairs of SNR measurements. The asterisks indicate statistically significant differences between the methods (*p* < 0.05). SPRING‐RIO TSE (2‐NSA) has the highest SNR in both WM and GM

Figure 8 shows a comparison of axial images acquired using the proposed SPRING‐RIO TSE, with one signal average (top row) and with two signal averages (middle row), and standard Cartesian TSE (bottom row). No obvious artifacts are observed in the SPRING‐RIO TSE images. The results indicate that the image quality of SPRING‐RIO TSE with 1‐NSA is, in general, comparable to that of Cartesian TSE, yet with only half of the scan time that is used for Cartesian TSE. With 2‐NSA, SPRING‐RIO TSE shows a higher SNR, and that with both 1‐NSA and 2‐NSA show similar or slightly better contrast than the Cartesian counterpart in some areas indicated by the yellow circles, such as the dentate nuclei, substantial nigra, and red nuclei. This is also demonstrated by the measured SNR in WM and GM with SPRING‐RIO TSE versus standard Cartesian TSE as shown in Figure 9, and image contrast between regions of iron deposition and surrounding tissue, and between GM and WM (see Supporting Information Figure S3).

**FIGURE 10:**
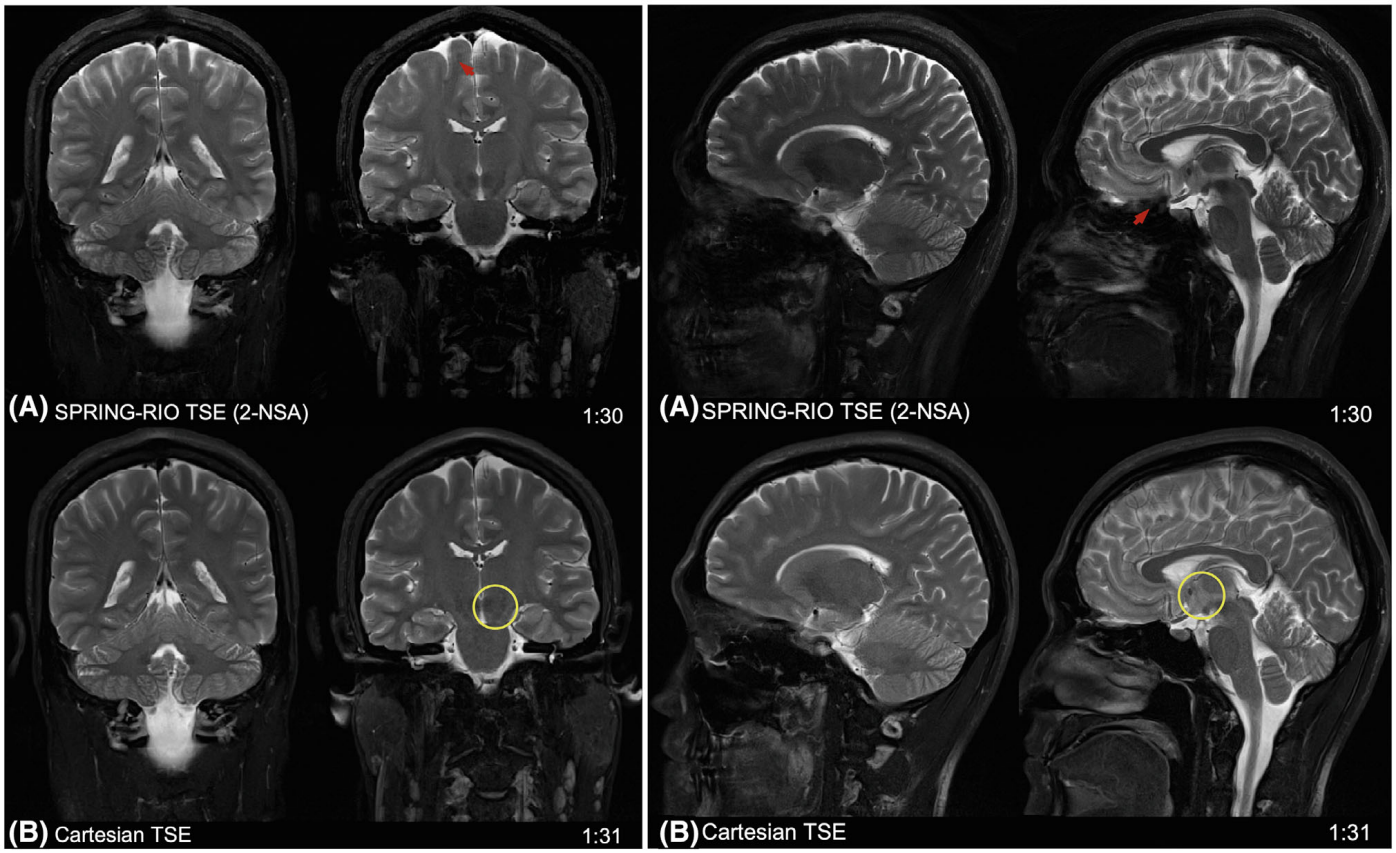
Comparison of in vivo sagittal and coronal images acquired using the proposed SPRING‐RIO TSE method and standard Cartesian TSE. The red arrows point to the structures where residual signal loss or artifacts exist, likely due to susceptibility or concomitant gradients. The yellow circles indicate areas where the image contrast is visually better in SPRING‐RIO TSE than in Cartesian TSE

For the sagittal and coronal data sets shown in Figure 10, residual signal loss and artifacts can be seen in some areas where there are strong susceptibility gradients, and ghosting artifacts, potentially induced by concomitant fields, are observed in frontal lobes, as indicated by red arrows. Nonetheless, the overall image quality of SPRING‐RIO TSE is comparable to that of Cartesian TSE, with improved contrast in areas with iron deposition (Figure 10, yellow circles).

## 4 DISCUSSION

TSE echo trains provide a means for maintaining signal pathways over a long acquisition window (∼200 ms) using a series of high‐flip‐angle refocusing RF pulses. However, the SAR limitations may restrict the protocol by limiting the number and the flip angle of refocusing RF pulses and the minimum spacing of the spin echoes, especially at high magnetic field strengths. In a typical Cartesian TSE protocol, the refocusing flip angle is reduced to $∼150^{{}^{\circ}}$ to mitigate the high SAR. For the SPRING‐RIO TSE protocol used in this work, the refocusing flip angle was set to the same value as that used in Cartesian TSE, while the SAR from the refocusing RF pulses is approximately 86% of that from Cartesian TSE, primarily due to a higher k‐space coverage per spin echo with a smaller number of echoes. Further reduction in SAR can be realized by optimizing the protocol. Flexibility exists to change the data acquisition time to allow a tradeoff between the ETL and off‐resonance artifact reduction. For a fixed set of parameters (e.g., FOV, in‐plane spatial resolution, and total scan time), increasing the readout duration leads to a shorter ETL, which results in a smaller number of refocusing RF pulses and thus a decreased SAR. For example, doubling the readout acquisition window from 7 ms to 14 ms results in an ETL of 7, which would result in a SAR value that is approximately 47% of that from Cartesian TSE, if the same refocusing flip angle was used. The influence of reduced refocusing flip angles and ETL on image contrast is beyond the scope of this preliminary study; future clinical studies are needed to evaluate these impacts on image quality and contrast.

Imaging speed is an important metric, and fast scanning is one of the advantages that SPRING‐RIO TSE provides. With the protocols used in this study, the minimum scan time of SPRING‐RIO TSE is roughly half of that used in Cartesian TSE (0:45 min/14 slices versus 1:31 min/14 slices). Li et al.^8^ proposed an alternative strategy to Cartesian TSE using an interleaved, rotated spiral‐in/out readout along with a double‐encoding method. However, the double‐encoding method requires additional scan time, reducing the imaging speed by half, and it may be more sensitive to any motion/flow artifacts. An abstract describing an interleaved, split spiral in‐out acquisition that alleviates T_2_‐decay effects without the need of a double‐encoding was recently reported in Ref. 32. Although in this work we did not compare SPRING‐RIO TSE to that technique, a future comparison of these two methods is planned. Our proposed method offers flexibility for fast scanning in tens of seconds, with a clinically acceptable SNR. Increasing the readout time (e.g., 10 ∼ 15 ms) or using a longer ETL is feasible to further improve scan efficiency, although it may induce stronger off‐resonance effects or an increased RF SAR. Incorporating non‐Cartesian parallel imaging techniques^33^, ^34^ can further accelerate the sampling speed, and it may be attractive for time‐limited applications, such as breath‐held single‐shot abdominal imaging.^35^

Gradient infidelity is one of the major concerns for reliable spiral readout imaging. The k‐space trajectories can be measured and incorporated into reconstruction to improve image quality, yet it is impractical to do that for every imaging slice and each sequence parameter set. In our implementation, a model‐based method that combines tuning the anisotropic delays on different gradient axes and eddy current compensation was used to estimate the actual k‐space trajectories. The calculated system parameters can be used for later scans after a one‐time gradient waveform calibration with no time penalty. This approach achieves good performance, as evidenced by the image quality of the phantom study.

Off‐resonance induced phase error is another concern for spiral imaging, especially for a long acquisition window. As observed from in vivo results, effective deblurring was accomplished in the majority of the images. The performance is sometimes suboptimal in two scenarios: (1) in areas with low amplitude or little contrast, such as in nearly uniform regions; and (2) if a local field fluctuates too rapidly, the objective function surface will produce erroneous extrema, because the conjugate phase reconstruction assumes a spatially smooth and temporally constant field map. This typically produces errors in areas around the sinus, nasal cavity, and mouth, where the anatomical structures in the spiral images are not as clean as those in the Cartesian images. Although a modest readout duration was used in this work to avoid large B_0_‐field induced phase accruals, and the affected areas are of little clinical significance, future work will optimize the deblurring method to deal with these challenges.

Concomitant (Maxwell) fields may cause phase errors as well, especially for spiral‐based TSE sequences, since spiral waveforms vary along the echo train, which may disturb the spin echo train.^36^, ^37^ Although Maxwell terms scale inversely with the field strength, and concomitant gradient effects decrease as the field strength increases, we still see a potential source of Maxwell field induced artifacts at 3T from SPRING‐RIO TSE in coronal and sagittal planes and off‐center slices. This work did not include concomitant‐gradient compensation; however, there are several correction methods for spiral TSE via gradient waveform redesign and/or phase correction during reconstruction.^8^, ^36^, ^37^, ^38^, ^39^ For example, the gradient waveform modifications presented by Mugler et al.^39^ have been incorporated into interleaved, rotated spiral TSE imaging with different trajectory types, and promising results show a substantial reduction in degradation associated with self‐squared Maxwell gradient effects at a low magnetic field strength (0.55T). This approach could be extended to the SPRING‐RIO TSE sequence to reduce the phase shifts at echoes and maintain the CPMG condition over echo spacings by gradient waveform modifications.

With a retraced in/out strategy, there is less flexibility to arbitrarily set the number of spiral rings, ETL, and FOV for a given resolution while targeting the desired TE because of their interdependencies. However, a target TE can still be approximately achieved by adjusting these imaging parameters and utilizing early echoes before the effective TE. For example, short‐TE images can be acquired by simply dropping one to two spiral‐in rings at the beginning with slightly reduced high frequency information.

## 5 CONCLUSIONS

We demonstrated that using annular spiral rings with a retraced in/out trajectory is a viable data acquisition method that can be incorporated into 2D TSE echo trains to efficiently suppress T_2_‐decay effects. With trajectory‐fidelity and off‐resonance corrections, this approach provides a potential alternative to Cartesian TSE for T_2_‐weighted neuroimaging, with high scan efficiency, low SAR, and improved image contrast.

## Supporting information


**Figure S1**. Difference images between the SPRING TSE (left), SPRING‐RIO TSE (right) and the reference. T_2_‐decay effect with T_2_ = 70 ms (top) and off‐resonance effect with a constant frequency offset of corresponding to 0.25, 0.5, and 0.75 cycles of phase (bottom) were simulated using a digital brain phantom.
**Figure S2**. Simulation results of one inferior slice with air/susceptibility from a digital brain phantom with off‐resonance effects for SPRING TSE and SPRING‐RIO TSE. Off‐resonance effects were simulated for three different amounts (1/4, 1/2, and 3/4 cycles) of phase accumulation. The image (bottom) with no phase accumulation was used as the reference, and SSIM values were calculated between the reconstructed images of each sequence and the reference. Compared to SPRING TSE, the artifacts and signal loss in SPRING‐RIO TSE are reduced and largely self‐corrected when off‐resonance is moderate (i and ii).
**Figure S3**. Measured contrast between RIOs. The first five groups (yellow regions 1 ∼ 5) measure the contrast between the areas with iron deposition and the surrounding tissue. The next four groups (blue regions 1 ∼ 4) measure the contrast between gray and white matter in the frontal lobe.
Table S1. Sequence parameters for SPRING TSE, SPRING‐RIO TSE, and Cartesian TSE.
Appendix A
Appendix B
