# Supplementary material for: SPRING‐RIO TSE: 2D T2‐Weighted Turbo Spin‐Echo brain imaging using SPiral RINGs with retraced in/out trajectories
Source: Magn Reson Med. 2022 Apr 8;88(2):601–16. doi: 10.1002/mrm.29210 (PMC9232877; doi:10.1002/mrm.29210)
Supplement: Supplementary file 1 — Figure S1. Difference images between the SPRING TSE (left), SPRING‐RIO TSE (right) and the reference. T2‐decay effect with T2 = 70 ms (top) and off‐resonance effect with a constant frequency offset of corresponding to 0.25, 0.5, and 0.75 cycles of phase (bottom) were simulated using a digital brain phantom. Figure S2. Simulation results of one inferior slice with air/susceptibility from a digital brain phantom with off‐resonance effects for SPRING TSE and SPRING‐RIO TSE. Off‐resonance effects were simulated for three different amounts (1/4, 1/2, and 3/4 cycles) of phase accumulation. The image (bottom) with no phase accumulation was used as the reference, and SSIM values were calculated between the reconstructed images of each sequence and the reference. Compared to SPRING TSE, the artifacts and signal loss in SPRING‐RIO TSE are reduced and largely self‐corrected when off‐resonance is moderate (i and ii). Figure S3. Measured contrast between RIOs. The first five groups (yellow regions 1 ∼ 5) measure the contrast between the areas with iron deposition and the surrounding tissue. The next four groups (blue regions 1 ∼ 4) measure the contrast between gray and white matter in the frontal lobe. Table S1. Sequence parameters for SPRING TSE, SPRING‐RIO TSE, and Cartesian TSE. Appendix A Appendix B [file MRM-88-601-s001.docx]

**Supporting Information**


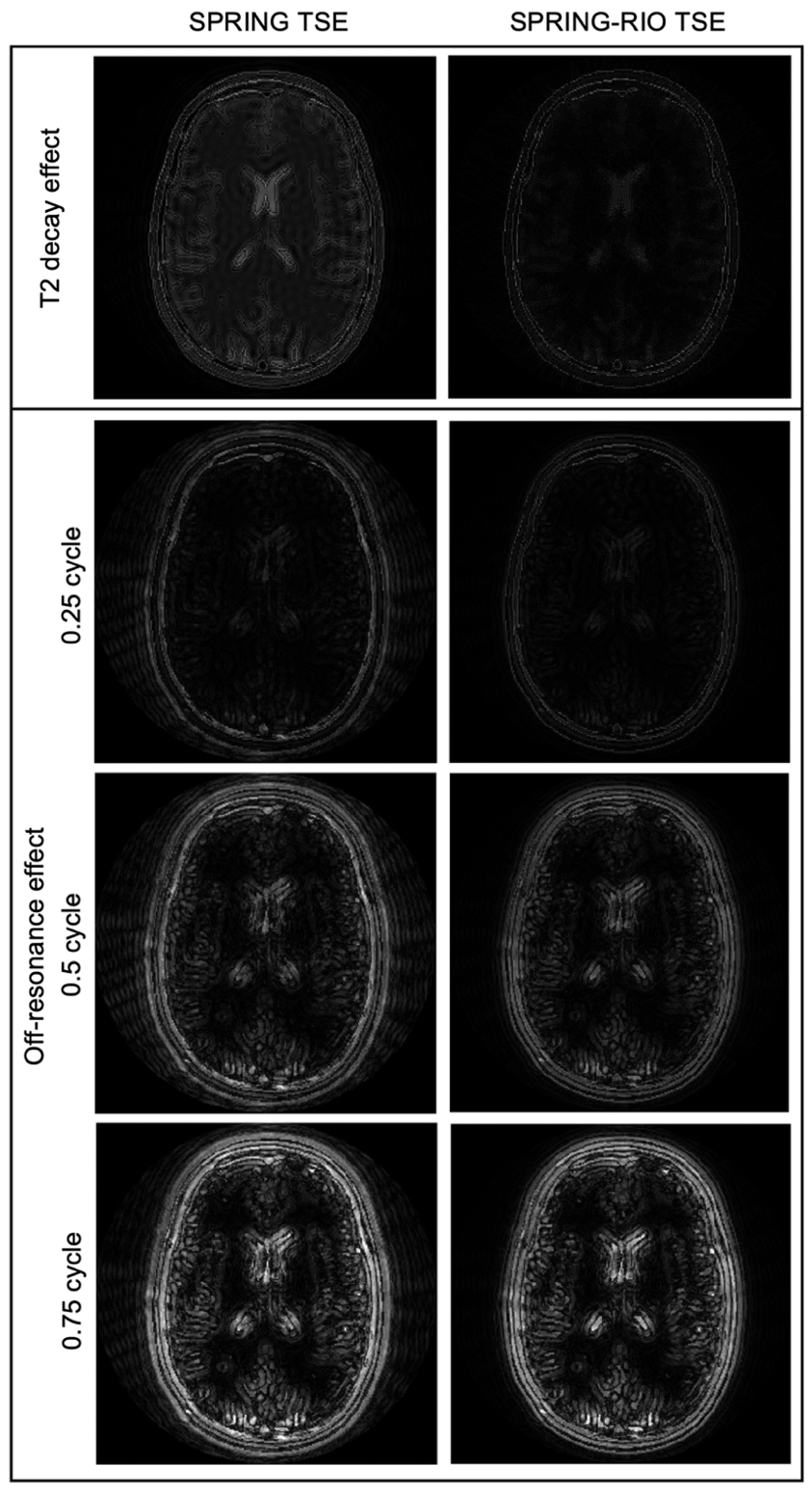


Supporting Information Figure S1. Difference images between the SPRING TSE (left), SPRING-RIO TSE (right) and the reference. T_2_-decay effect with T_2_ = 70 ms (top) and off-resonance effect with a constant frequency offset of corresponding to 0.25, 0.5, and 0.75 cycles of phase (bottom) were simulated using a digital brain phantom.

*
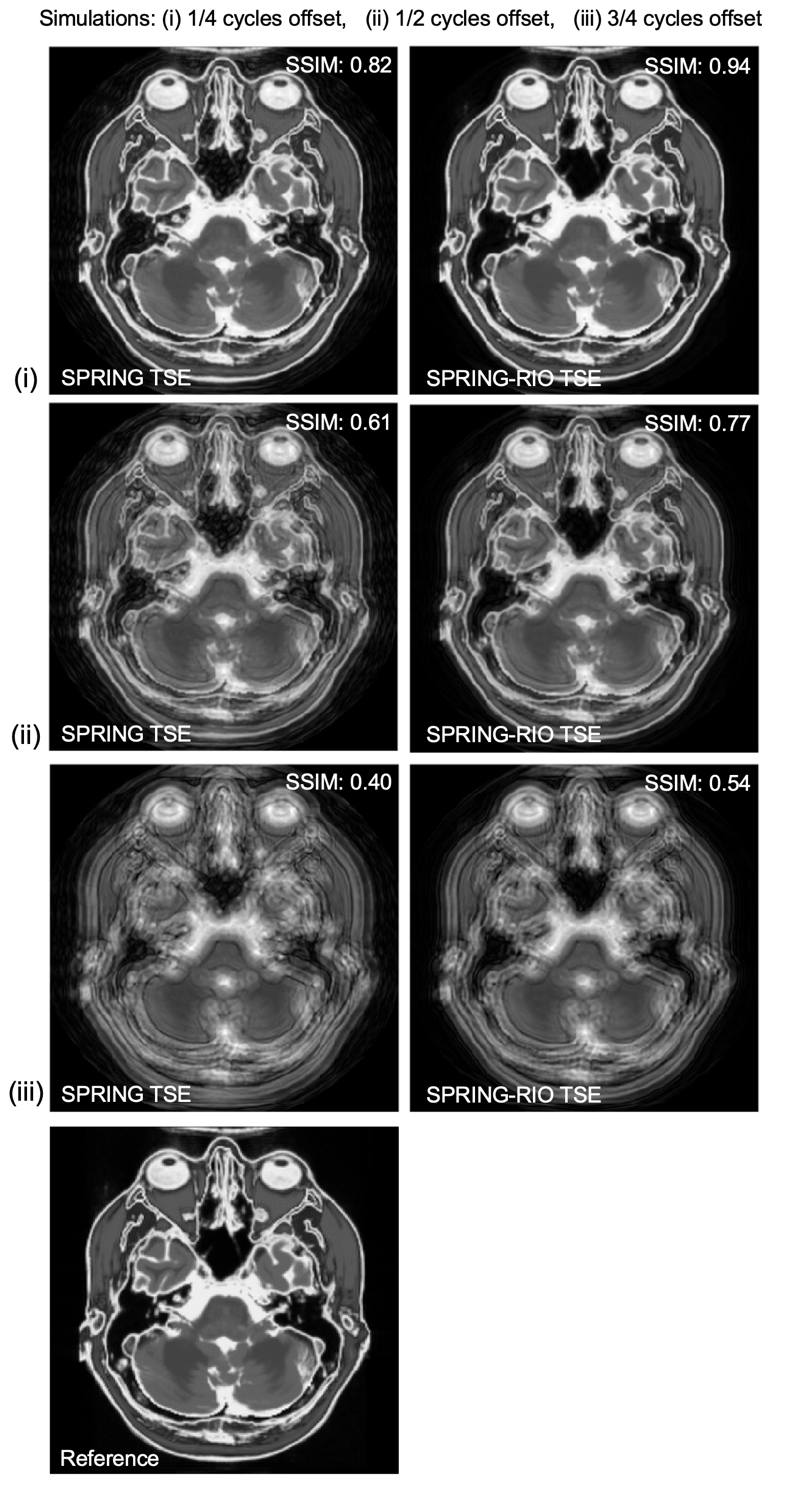
*

Supporting Information Figure S2. Simulation results of one inferior slice with air/susceptibility from a digital brain phantom with off-resonance effects for SPRING TSE and SPRING-RIO TSE. Off-resonance effects were simulated for three different amounts (1/4, 1/2, and 3/4 cycles) of phase accumulation. The image (bottom) with no phase accumulation was used as the reference, and SSIM values were calculated between the reconstructed images of each sequence and the reference. Compared to SPRING TSE, the artifacts and signal loss in SPRING-RIO TSE are reduced and largely self-corrected when off-resonance is moderate (i and ii).


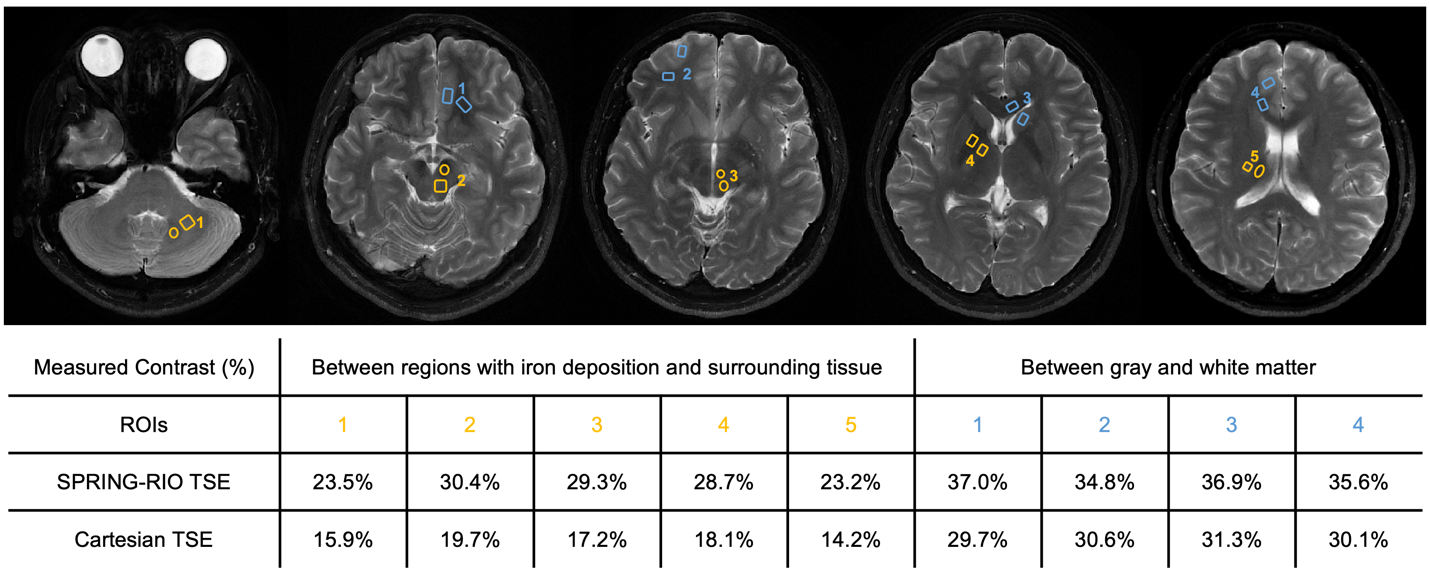


Supporting Information Figure S3. Measured contrast between RIOs. The first five groups (yellow regions 1~5) measure the contrast between the areas with iron deposition and the surrounding tissue. The next four groups (blue regions 1~4) measure the contrast between gray and white matter in the frontal lobe.

*
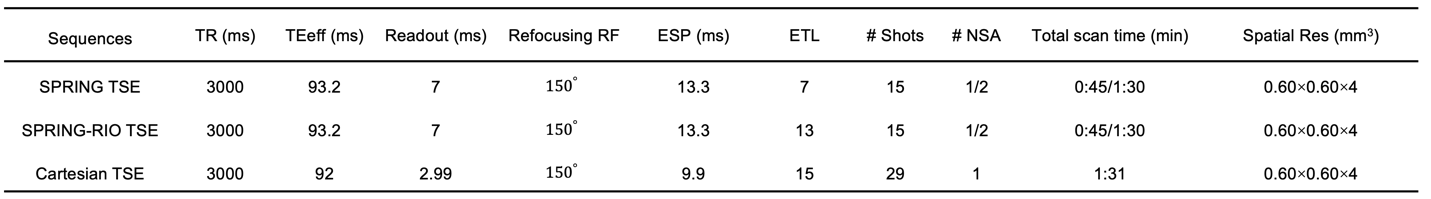
*

Supporting Information Table S1. Sequence parameters for SPRING TSE, SPRING-RIO TSE, and Cartesian TSE.

**Appendix A**

The received MR signal of a spiral-out ring for the $j^{th}$ k-space segment acquired at the $p^{th}$ echo can be modeled for $t\in[-\frac{T}{2},\frac{T}{2}]$ as

$s_{j,p}\left( t \right)=\int m\left( \boldsymbol{r} \right)e^{-i2\pi\boldsymbol{k}_{\boldsymbol{j,p}}\left( t \right)\boldsymbol{r}}e^{-i\omega\left( r \right)t}e^{\frac{-(t+T_{p})}{T_{2}}}d\boldsymbol{r}$**,**

where $T$ is the readout time, and $m(\boldsymbol{r})$ is the object’s complex-valued magnetization. $\omega(\boldsymbol{r})$ is defined as the spatially varying resonant frequency of the object. $T_{p}$ refers to the time interval between the excitation RF pulse and the center of the readout window at $p^{th}$ echo, where$0\leq p\leq L-1$, and $L$ is the total number of spiral-out rings.

Using the similar substitution, the received MR signal of a second acquisition from a spiral-in ring for the same $j^{th}$ k-space segment but acquired at the $q^{th}$ echo can be derived as, for $t'\in[-\frac{T}{2},\frac{T}{2}]$:

$s_{j,q}\left( t' \right)=\int m(\boldsymbol{r})e^{-i2\pi\boldsymbol{k}_{\boldsymbol{j,q}}\left( t^{'} \right)\boldsymbol{r}}e^{-i\omega(\boldsymbol{r})t^{'}}e^{\frac{-(t^{'}+T_{q})}{T_{2}}}d\boldsymbol{r}$**,**

where $T_{q}$ refers to the time interval between the excitation RF pulse and the center of the readout window at $q^{th}$ echo, and $-L+1\leq q\leq0$.

For retraced in-out trajectories, a few properties of the k-space trajectory must be defined. First, each ring segment has duration $T$, and each pair of the two retraced rings are anti-symmetric about the echo time such that

$T_{p}+T_{q}=2 TE$.

Then, we can constrain the ring trajectories to be time-reversed copies of each other as

$\boldsymbol{k}_{\boldsymbol{j,p}}\left( t \right)=\boldsymbol{k}_{\boldsymbol{j,q}}\left( -t \right)=\boldsymbol{-k}_{\boldsymbol{j,q}}\left( t \right)$.

The central self-retraced spiral in-out segment can be considered as a special case when $p=q=0$. We can also define $\Delta T$ in terms of $T_{p},T_{q}$ as

$\Delta T= T_{p}- T_{q}$.

Assuming each readout in the TSE echo train is short relative to the T_2_ relaxation, we can write that

$e^{\frac{-T}{T_{2}}}\approx1$.

Before we can combine $s_{j,p}$ with $s_{j,q}$, we must time-reverse $s_{j,q}$ since the second spiral-in acquisition is run in the opposite direction through k-space. Let $t=-t'$, we can get

$s_{j,q}\left( -t \right)=\int m(\boldsymbol{r})e^{-i2\pi\boldsymbol{k}_{\boldsymbol{j,q}}\left( -t \right)\boldsymbol{r}}e^{-i\omega(\boldsymbol{r})(-t)}e^{\frac{-(-t+T_{q})}{T_{2}}}d\boldsymbol{r}$**.**

Using all the above assumptions, $s_{j,q}\left( -t_{2} \right)$ can be written as

$s_{j,q}\left( -t \right)\approx\int m(\boldsymbol{r})e^{-i2\pi\boldsymbol{k}_{\boldsymbol{j,p}}\left( t \right)\boldsymbol{r}}e^{i\omega(\boldsymbol{r})t}e^{\frac{{-(T}_{p}-\Delta T)}{T_{2}}}d\boldsymbol{r}$**.**

Now, the combination of the two signals can be written as

$$s\left( t \right)= \frac{s_{j,p}\left( t \right)+s_{j,q}\left( -t \right)}{2}$$

$=\frac{1}{2}\int M(\boldsymbol{r})e^{-i2\pi\boldsymbol{k}_{\boldsymbol{j,p}}\left( t \right)\boldsymbol{r}}e^{-\frac{T_{p}}{T_{2}}}\left[ e^{-i\omega(\boldsymbol{r})t}+e^{i\omega(\boldsymbol{r})t}e^{+\frac{\Delta T}{T_{2}}} \right]dr$.

Now, to simplify more:

$s\left( t \right)=\frac{1}{2}\int M(\boldsymbol{r})e^{-i2\pi\boldsymbol{k}_{\boldsymbol{j,p}}\left( t \right)\boldsymbol{r}}e^{\frac{-(T_{p}-\frac{\Delta T}{2})}{T_{2}}}\left[ e^{-i\omega(\boldsymbol{r})t}e^{-\frac{\Delta T}{{2T}_{2}}}+e^{i\omega(\boldsymbol{r})t}e^{\frac{\Delta T}{{2T}_{2}}} \right]dr$.

For the next step, substitute the terms $e^{i\omega(\boldsymbol{r})t}=\cos[\omega(\boldsymbol{r})t]+isin[\omega(\boldsymbol{r})t]$, $e^{\frac{\Delta T}{{2T}_{2}}}=\cosh\left( \frac{\Delta T}{{2T}_{2}} \right)+sinh(\frac{\Delta T}{{2T}_{2}})$, $e^{\frac{-\Delta T}{{2T}_{2}}}=$ $\cosh\left( \frac{\Delta T}{{2T}_{2}} \right)-sinh(\frac{\Delta T}{{2T}_{2}})$, $T_{p}-\frac{\Delta T}{2}=TE$, and $\frac{\Delta T}{2}= T_{p}-TE$.

Multiplying out the terms in the brackets, we can get

$s\left( t \right)=\int m\left( \boldsymbol{r} \right)e^{-i2\pi\boldsymbol{k}_{\boldsymbol{j,p}}\left( t \right)\boldsymbol{r}}e^{\frac{-TE}{T_{2}}}\left[ \cos[\omega(\boldsymbol{r})t]\cosh\left( \frac{T_{p}-TE}{T_{2}} \right)+isin[\omega(\boldsymbol{r})t]sinh(\frac{T_{p}-TE}{T_{2}}) \right]d\boldsymbol{r}$**.**

The equation above describes the magnitude and phase modulation of the combination of the signals collected from SPRING-RIO TSE due to the off-resonance effect during the readout window and T_2_-decay effect along the echo train.

**Appendix B**

We extend the original signal equation to conjugate phase reconstruction. Let

$\tilde{s}\left( t;\omega_{i}(\boldsymbol{r}) \right)= \frac{s_{j,p}\left( t \right)e^{i\omega_{i}(\boldsymbol{r})t}+s_{j,q}\left( -t \right)e^{-i\omega_{i}(\boldsymbol{r})t}}{2}$.

In this case, each ring trajectory is demodulated at frequency $\omega_{i}$, and the time reversal of $s_{j,q}$ produces a demodulation term that is the complex conjugate of that applied to $s_{j,p}$.

To evaluate $\tilde{s}\left( t;\omega_{i}(\boldsymbol{r}) \right)$ for a point object at location $\boldsymbol{r}_{\boldsymbol{o}}$,

$$\tilde{s}\left( t;\omega_{i} \right)|_{r_{o}}= e^{-i2\pi\boldsymbol{k}_{\boldsymbol{j,p}}\left( t \right)\boldsymbol{r}_{\boldsymbol{0}}.}e^{\frac{-TE}{T_{2}}}$$

$\left[ \cos\{[\omega\left( \boldsymbol{r}_{\boldsymbol{0}} \right)-\omega_{i}]t\}\cosh\left( \frac{T_{p}-TE}{T_{2}} \right)+isin\{\left[ \omega\left( \boldsymbol{r}_{\boldsymbol{0}} \right)-\omega_{i} \right]t\}\sinh(\frac{T_{p}-TE}{T_{2}}) \right]$.

We then multiply $\tilde{s}\left( t;\omega_{i} \right)$ by its complex conjugate and substitute ${cosh}^{2}\left( x \right)=1+{sinh}^{2}\left( x \right)$ to yield

$\tilde{s}\left( t;\omega_{i} \right)|_{r_{o}}{\tilde{s}\left( t;\omega_{i} \right)|_{r_{o}}}^{*}=e^{\frac{-TE}{T_{2}}}\left[ {cos}^{2}\left\{ \left[ \omega\left( \boldsymbol{r}_{\boldsymbol{0}} \right)-\omega_{i} \right]t \right\}+{sinh}^{2}\left( \frac{T_{p}-TE}{T_{2}} \right) \right]$.

The ${cos}^{2}\left\{ \left[ \omega\left( \boldsymbol{r}_{\boldsymbol{0}} \right)-\omega_{i} \right]t \right\}$ term attenuates the distribution of energy across the k-space trajectory for any $\omega_{i}\neq\omega\left( \boldsymbol{r}_{\boldsymbol{0}} \right)$. By invoking Parseval’s theorem, the integrated squared magnitude of the PSF is maximized at $\omega_{i}=\omega\left( \boldsymbol{r}_{\boldsymbol{0}} \right)$ for given sequence parameters. This final set shows that the magnitude modulation of the k-space energy caused by off-resonance is independent of T_2_-decay and incidental image phase.
